# Loss of 5α-Reductase Type 1 Accelerates the Development of Hepatic Steatosis but Protects Against Hepatocellular Carcinoma in Male Mice

**DOI:** 10.1210/en.2013-1592

**Published:** 2013-09-30

**Authors:** Joanna K. Dowman, Laurence J. Hopkins, Gary M. Reynolds, Matthew J. Armstrong, Maryam Nasiri, Nikolaos Nikolaou, E. Leonie A. F. van Houten, Jenny A. Visser, Stuart A. Morgan, Gareth G. Lavery, Andrei Oprescu, Stefan G. Hübscher, Philip N. Newsome, Jeremy W. Tomlinson

**Affiliations:** National Institute for Health Research Biomedical Research Unit and Centre for Liver Research (J.K.D., L.J.H., G.M.R., M.J.A., S.G.H., P.N.N.) and Centre for Endocrinology, Diabetes, and Metabolism (M.N., N.N., S.A.M., G.G.L., A.O., J.W.T.), University of Birmingham, Birmingham, United Kingdom; The Liver Unit (J.K.D., M.J.A., P.N.N.) and Department of Cellular Pathology (S.G.H.), Queen Elizabeth Hospital Birmingham, Edgbaston, Birmingham B15 2TT, United Kingdom; and Department of Internal Medicine (E.L.A.F.v.H., J.A.V.), Erasmus MC, 3000 CA Rotterdam, The Netherlands

## Abstract

Nonalcoholic fatty liver disease (NAFLD) has been associated with glucocorticoid excess and androgen deficiency, yet in the majority of patients with steatohepatitis, circulating cortisol and androgen levels are normal. The enzyme 5α-reductase (5αR) has a critical role in androgen and glucocorticoid action. We hypothesize that 5αR has an important role in the pathogenesis of steatohepatitis through regulation of intracrine/paracrine hormone availability. Human liver samples from patients with NAFLD and normal donor tissue were used for gene expression and immunohistochemical analysis. NAFLD samples were scored using the Kleiner classification. In addition, 5αR1^−/−^, 5αR2^−/−^, and wild-type (WT) mice were fed normal chow or American lifestyle-induced obesity syndrome (ALIOS) diet for 6 or 12 months. Liver histology was graded and staged. Hepatic and circulating free fatty acid and triglyceride levels were quantified, and gene and protein expression was measured by real-time PCR and immunohistochemistry. 5αR1 and -2 were highly expressed in human liver, and 5αR1 protein expression increased with severity of NAFLD. 5αR1^−/−^ (but not 5αR2^−/−^) mice fed an ALIOS diet developed greater hepatic steatosis than WT mice, and hepatic mRNA expression of genes involved in insulin signaling was decreased. Furthermore, 60% of WT mice developed focal hepatocellular lesions consistent with hepatocellular carcinoma after 12 months of the ALIOS diet, compared with 20% of 5αR2^−/−^ and 0% of 5αR1^−/−^ mice (*P* < .05). 5αR1 deletion accelerates the development of hepatic steatosis but may protect against the development of NAFLD-related hepatocellular neoplasia and therefore has potential as a therapeutic target.

Nonalcoholic fatty liver disease (NAFLD) encompasses a spectrum of disease from simple steatosis, through nonalcoholic steatohepatitis (NASH), to fibrosis and cirrhosis, with a risk of hepatocellular carcinoma (HCC) (1, 2). The recent rise in global levels of obesity and type 2 diabetes has resulted in a parallel increase in the prevalence of NAFLD such that it now represents the most common cause of liver disease in western countries (3, 4). Simple steatosis has a relatively benign prognosis (5), although the presence of NASH and/or fibrosis is associated with increased all-cause and liver-related morbidity and mortality (6, 7). Although our understanding of the pathogenic mechanisms underpinning the development and progression of NAFLD has increased significantly in recent years (8), efficacious disease-modifying therapies are lacking and current treatment strategies are largely aimed at cardiovascular and metabolic risk reduction (9).

Both glucocorticoids (GCs) and androgens have been implicated in the pathogenesis of NAFLD. Patients with GC excess, Cushing's syndrome, develop a reversible phenotype characterized by central obesity, insulin resistance, hypertension, glucose intolerance, and in some cases NAFLD (10). In contrast, androgen deficiency and low circulating testosterone levels are also associated with the development of NAFLD (10–13). However, in the vast majority of patients with NAFLD, circulating GC and androgen levels are normal.

The isoforms of 5α-reductase (5αR) are crucial in both GC and androgen metabolism. They convert GCs (including cortisol and corticosterone) to their less active dihydrometabolites with subsequent conversion to tetra-hydrometabolites by 3α-hydroxysteroid dehydrogenase. In addition, they catalyze the conversion of testosterone to the more potent androgen, DHT (14), which has led to the use of 5αR inhibitors for the treatment of benign prostatic hyperplasia and other androgen-driven diseases.

Three isoforms of 5αR have been identified; 5αR1 and -2 have an established role in androgen metabolism (10, 15), but the functional significance of the recently identified 5αR3 remains to be determined, and its true role is likely to be in posttranscriptional glycosylation (16). 5αR1 and -2 are located within the endoplasmic reticulum and share less than 50% homology, and the genes that encode them are located on different chromosomes (5 and 2, respectively). 5αR2 is highly expressed in the prostate and urogenital epithelium, and mutations lead to 46XY disorders of sexual development (17).

The 5αRs have broad substrate specificities and are able to reduce the A-ring of many steroids including progesterone, androstenedione, and corticosterone as well as cortisol and testosterone. Detailed enzyme kinetics and substrate specificities have been performed in cell lysates and suggest that androgens and progesterone rather than GCs are the preferred substrates (14, 15).

The role of 5αR in the pathogenesis of NAFLD has not been explored, although patients with insulin resistance (18) and NAFLD (19, 20) have increased 5αR activity. Crucially, both isoforms are highly expressed in human liver; however, in rodent liver, there is little or no expression of 5αR2, although it is expressed in other tissues (15).

The American lifestyle-induced obesity syndrome (ALIOS) murine model of NAFLD reflects the characteristics of the American fast-food diet with a high trans-fatty acid content, and supplementary high-fructose corn syrup (21), both of which are believed to be important in the pathogenesis of NAFLD (22–24). We have previously shown that mice fed the ALIOS diet for up to 12 months develop glucose intolerance, obesity, and insulin resistance as well as inflammatory NASH with advanced (F3) fibrosis (25). In addition, mice develop HCC in a significant proportion of cases (8). Using this model in mice with genetic deletion of 5αR1 and -2 as well as normal and diseased explanted human samples, we tested the hypothesis that decreased 5αR activity may accelerate the onset and progression of NAFLD.

## Materials and Methods

### Human tissue studies

Liver explant tissues were obtained from the liver transplantation program at Queen Elizabeth Hospital, Birmingham, United Kingdom. Ethical approval and patient consent were obtained before collection. The 6-μm acetone-fixed frozen sections were stained with hematoxylin and eosin (H&E) to examine morphology and hematoxylin van Gieson for fibrosis. Specimens were classified as normal (n = 6), steatosis alone (n = 3), NASH without advanced fibrosis (F0–F3, n = 4), and NASH cirrhosis (F4, n = 15). Each specimen was also graded and staged according to the Kleiner scoring system by an expert liver pathologist (S.G.H.) to produce an NAFLD activity score (NAS). The Kleiner system generates a composite score based on the degree of steatosis (0–3), lobular inflammation (0–3), and hepatocyte ballooning (0–2), with a separate score for fibrosis (0–4) (26).

### RNA isolation, reverse transcription, and real-time PCR

RNA was isolated from frozen human and rodent (see below) liver tissue using the QIAGEN RNEasy MiniKit (QIAGEN 74104) according to a standard protocol, and RNA concentration was measured by spectrophotometry (NanoDrop Technologies, Labtech International). Total RNA (1 μg) was denatured with 200 ng of random primers in a volume of 10 μL, and 20 U avian myeloblastosis virus, 20 U ribonuclease inhibitor, 1μM deoxy-NTPs and 5× reaction buffer were added to the volume of 20 μL. The reverse transcription reaction was carried out at 37°C for 1 hour. The reaction was terminated by heating the cDNA to 95°C for 5 minutes. Real-time PCR using TaqMan 20× gene expression assays (Applied Biosystems) were used to measure mRNA expression levels and normalized to *18S* RNA as internal control. Data are expressed as cycle threshold (Ct) values (Ct = cycle number at which logarithmic PCR plots cross a calculated threshold line) and used to determine ΔCt values (ΔCt = Ct of the target gene − Ct of the housekeeping gene). Fold changes were calculated using the transformation (fold increase = 2^−ΔΔCt^), and data are expressed as arbitrary units (AU) using the following transformation: expression = 1000 × (2^−ΔCt^) AU. The ΔCt values were used for statistical analyses.

### Immunohistochemistry

Immunohistochemistry was performed using rabbit polyclonal antibodies directed against the human steroid 5αR1 and 5αR2 isozymes (kindly donated by Professor David Russell, University of Texas Southwestern Medical Center, Dallas, Texas) (Supplemental Table 1, published on The Endocrine Society's Journals Online website at http://endo.endojournals.org). Immunohistochemistry was performed using the ImmPRESS peroxidase universal antimouse/antirabbit Ig reagent kit (Vector MP-7500) and visualized with diaminobenzidine. Two independent blinded reviewers (J.K.D. and M.J.A.) performed a semiquantitative assessment of expression based on both the distribution and intensity of hepatocyte staining in each section. Total score (ranging from 0–12) = distribution (0, no expression; 1, expression in <5% of hepatocytes; 2, expression in 5 to <33% of hepatocytes; 3, expression in 33%–66% of hepatocytes; 4, expression in >66% of hepatocytes) × intensity (0, no expression; 1, mild expression; 2, moderate expression; 3, strong expression).

### Rodent studies

Male C57BL/6 × 129/Sv mice aged 6 to 8 weeks were housed in accordance with animal care protocols at the University of Birmingham. Mice were maintained on a 12-hour light, 12-hour dark schedule at 22°C, with up to 4 mice per cage. Individual animals were weighed weekly. Mice were fed either an ALIOS diet (containing 45% kcal from fat, 37% from carbohydrate, and 18% from protein) (TD.06303; Harlan Teklad) (21) or normal chow (NC). Mice fed the ALIOS diet had their drinking water replaced by a high-fructose corn syrup equivalent, prepared by adding 42 mL/L of a 55% fructose and 45% glucose solution. Food and drink was provided ad libitum to both ALIOS and control animals.

Male 5αR1^−/−^ and 5αR2^−/−^ mice were kindly provided by Dr Mala Mahendroo (University of Texas Southwestern). Heterozygote × heterozygote breeding was used to generate homozygous knockout mice as well as wild-type (WT) control mice. In total, 100 mice were studied; 50 WT, 25 5αR1^−/−^, and 25 5αR2^−/−^ mice were examined at baseline or fed either ALIOS or NC diet for both 6 and 12 months before culling and tissue analysis. Mice were killed by cervical dislocation under terminal general anesthesia using isoflurane before organ retrieval. Genotyping was performed using QIAGEN DNeasy blood and tissue kit (no. 69506) according to the manufacturer's instructions. Confirmatory genotyping was repeated after completion of experiments.

### Corticosterone- and DHT-treated rodent studies

The 6-week old male WT C57BL/6J mice were treated with corticosterone (100 μg/mL, 0.66% ethanol) or vehicle (0.66% ethanol) via drinking water for 5 weeks (27). Water was replaced twice weekly. Before mice were killed, they were fasted for 4 hours and then culled by cervical dislocation and liver tissue harvested and snap frozen in liquid nitrogen.

C57BL/6J WT female mice were obtained from the Animal Facility of the Erasmus Medical Center and were kept under standard animal housing conditions in accordance with the National Institutes of Health Guidelines for the Care and Use of Experimental Animals. The experiments were performed with permission of the local ethics committee. The 3-week old mice of similar body weight were implanted with either an sc 90-day continuous-release DHT pellet (Innovative Research of America) (total dose, 2.5 mg; daily dose, 27.5 μg) or placebo. Mice were culled at the end of the treatment period (90 days) and liver tissue harvested as described above (28).

### Glucose tolerance testing

Glucose tolerance was measured by ip glucose tolerance test (GTT) 5 to 14 days before culling. Mice were fasted for 5 hours before baseline tail vein blood glucose testing using a portable glucometer (Accuchek Aviva; Roche). Tail vein blood glucose levels were then measured at 15, 30, 60, and 120 minutes after ip injection of 1 mg/kg of a sterile 20% dextrose solution.

### Free fatty acid and triglyceride quantification

Commercial colorimetric assays were used for free fatty acid (FFA) quantification in serum and whole tissue (Biovision K612–100) and for tissue triglyceride quantification (Biovision K622–100) according to the manufacturer's instructions. All samples were analyzed in duplicate. Measurement of tissue FFA was performed after chloroform extraction of lipids from 10-mg snap-frozen whole liver tissue samples. Quantification of FFA in serum was performed using 6-μL snap-frozen serum samples diluted to 5 0μL with assay buffer.

### Histological analysis, gene expression, and serum profiling

Formalin-fixed liver sections were embedded in paraffin and sectioned (4 μm). Sections were stained with H&E and hematoxylin van Gieson and then graded and staged according to the Kleiner system by an expert liver pathologist (S.G.H.) blinded to diet and genotype as described above. Immunohistochemistry was performed as described above. Antibodies against α-smooth muscle actin (α-SMA) (Abcam [E184] ab32575), Ki67 (Millipore AB9260), androgen receptor (AR) (Millipore PG-21), wide-spectrum cytokeratin (pan-CK; Dako Z0622), and (SRY [sex determining region Y]-box 9) Sox9) (Millipore AB5535) were used (Supplemental Table 1). α-SMA and pan-CK staining was quantified by measuring the proportion of surface area stained using image analysis software. Ki67 and Sox9 staining was quantified by counting the mean number of positively stained parenchymal cells in 5 nonoverlapping fields at ×200 magnification.

Hepatic gene expression analysis was performed using the Fluidigm 96 reactions Dynamic Array (Applied Biosystems) according to the manufacturer's protocol. All rodent primers were obtained from Applied Biosystems. Changes in mRNA expression were calculated using difference of Ct values and normalized to the housekeeping gene cyclophilin A. The ΔCt and fold changes as well as statistical analysis were performed as described for human samples.

Liver biochemistry was measured using standard laboratory methods (Roche modular system).

### Cell culture studies

The C3A human hepatocyte cell line was purchased from LGC Standards (American type culture collection CRL-10741) and cultured in Eagle's MEM containing 10% fetal calf serum and glutamine/penicillin/streptococcus. Lipogenesis was measured by the uptake of 1-[^14^C]acetate into the lipid component as described previously (29). Briefly, at confluence, cells were washed and cultured for 4 hours in serum-free medium and then incubated in 500 μL serum-free medium in the presence or absence of cortisol (250nM), Testosterone (50nM) or DHT (10nM) for 18 hours. 1-[^14^C]Acetic acid (0.12 μCi/L) with cold sodium acetate (10μM) was added to the treatments for an additional 6 hours (total treatment time is therefore 24 hours). After incubation, cells were washed and scraped into 250 μL PBS. The lipid fraction was recovered in Folch solvent, the solvent was evaporated, and the radioactivity retained in the cellular lipid was determined by scintillation counting.

### Statistical analysis

Statistical analyses were performed using GraphPad Prism version 5. Comparison of 2 variables was performed using Student's *t* test or Mann-Whitney *U* test for parametric and nonparametric data, respectively. A χ^2^ analysis was used to compare the frequency of specific observations (actual vs expected). For studies involving multiple comparisons, one-way ANOVA with Bonferroni's multiple-comparison test or Kruskal-Wallis with Dunn's multiple-correction tests were used to determine statistical significance. Two-tailed significance was *P* < .05.

## Results

### 5αR expression in human liver and NAFLD

**Figure 1.**
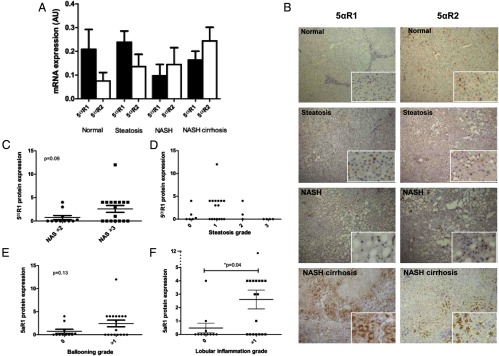
A, 5αR1 and 5αR2 mRNA expression in human liver biopsies does not change across the NAFLD spectrum. B, However, immunohistochemical staining demonstrates increased 5αR1 (but not 5αR2) protein levels with increasing severity of NAFLD. C–F, 5αR1 protein levels are not associated with NAS (C), steatosis grade (D), and hepatocyte ballooning (E), but expression is highest in those biopsies with the most lobular inflammation (F).

5αR1 and 5αR2 mRNA were detected in all human liver samples; however, gene expression was not different in samples from patients with NAFLD compared with normal liver (Figure 1A). Immunohistochemical staining demonstrated that the 5αR isoforms were expressed in a predominantly parenchymal distribution. 5αR1 expression increased with histological severity of NASH (Figure 1B). 5αR2 was also expressed, although this did not change across the histological spectrum of NAFLD (Figure 1B). Although 5αR1 protein levels were increased in those samples with the highest NASs and those with evidence of hepatocyte ballooning, these failed to reach statistical significance (Figure 1, C–E). However, 5αR1 protein levels increased with lobular inflammation (*P* < .05) (Figure 1F). No significant associations were observed between 5αR2 expression and NAS or any of its individual components or between either 5αR isoform and fibrosis stage.

### 5αR deletion and the pathogenesis of NAFLD

To determine whether manipulation of 5αR activity may regulate the pathogenesis of NAFLD, studies were conducted in 5αR1^−/−^ and 5αR2^−/−^ mice fed either NC or ALIOS diet for up to 12 months.

### Body weight and glucose tolerance in 5αR1^−/−^ and 5αR2^−/−^ mice

Body weight, fasting blood glucose, and glucose area under the curve after ip GTT were not significantly different in WT, 5αR1^−/−^, and 5αR2^−/−^ mice either at baseline or at 6 or 12 months of NC feeding (Table 1). Although 5αR2^−/−^ mice were heavier than their WT littermates after 6 months of the ALIOS diet, there were no significant differences in body weight or glucose tolerance between WT, 5αR1^−/−^, and 5αR2^−/−^ mice at any other time point (Table 2). There were no differences in the patterns of glucose disposal across the ip GTT between WT and 5αR1^−/−^ and 5αR2^−/−^ mice.

**Table 1.** Body Weights, LBWR, GTT Values, Serum Transaminase Levels, Circulating and Hepatic FFA Levels, and α-SMA Expression in WT, 5αR1^−/−^, and 5αR2^−/−^ Mice Fed NC Diet for 6 or 12 Months^a^

	6 Months		12 Months		6 Months		12 Months	
	WT	5αR1^−/−^	WT	5αR1^−/−^	WT	5αR2^−/−^	WT	5αR2^−/−^
Body weight, g	33.8 ± 1.6	34.4 ± 2.1	43.5 ± 2.7	47.3 ± 2.9	35.20 ± 1.8	36.3 ± 1.4	43.8 ± 1.8	41.5 ± 2.6
LBWR	0.04 ± 0.00	0.04 ± 0.00	0.04 ± 0.00	0.05 ± 0.00	0.05 ± 0.00	0.04 ± 0.00	0.05 ± 0.00	0.05 ± 0.00
Fasting glucose, mmol/L	9.1 ± 0.9	7.9 ± 0.4	9.3 ± 1.0	8.1 ± 0.7	8.8 ± 0.7	8.6 ± 0.5	8.3 ± 1.0	8.9 ± 0.6
2 h glucose, mmol/L	12.3 ± 3.2	8.8 ± 0.6	18.4 ± 4.6	15.2 ± 3.9	10.4 ± 1.3	12.5 ± 0.5	20.7 ± 4.4	18.9 ± 2.9
GTT AUC, mmol/L·h	635 ± 149	422 ± 72	2491 ± 439	2127 ± 402	464 ± 112	1035 ± 119	2338 ± 463	1523 ± 357
AST, IU/L	169 ± 82	116 ± 61	176 ± 97	97 ± 13	55 ± 11	55 ± 6	78 ± 11	71 ± 13
ALT, IU/L	44 ± 10	39 ± 8	102 ± 46	81 ± 12	27 ± 3	34 ± 5	66 ± 20	43 ± 5
Circulating FFA, nmol/μL	0.46 ± 0.05	0.53 ± 0.11	0.65 ± 0.06	0.68 ± 0.05	0.49 ± 0.03	0.46 ± 0.10	0.42 ± 0.07	0.57 ± 0.03
Hepatic FFA, nmol/mg	1.89 ± 0.57	2.14 ± 0.14	2.05 ± 0.39	1.63 ± 0.18				
αSMA, % positive area			0.58 ± 0.22	0.61 ± 0.22			0.34 ± 0.05	0.39 ± 0.12

Abbreviation: AUC, area under the curve.

a All data are expressed as mean ± SEM.

**Table 2.** Body Weights, LBWR, GTT Values, Serum Transaminase Levels, Circulating and Hepatic FFA Levels, and α-SMA Expression in WT, 5αR1^−/−^, and 5αR2^−/−^ Mice Fed ALIOS Diet for 6 or 12 Months^a^

	6 Months		12 Months		6 Months		12 Months	
	WT	5αR1^−/−^	WT	5αR1^−/−^	WT	5αR2^−/−^	WT	5αR2^−/−^
Body weight, g	38.5 ± 3.6	42.6 ± 1.4	46.2 ± 3.9	43.7 ± 1.6	37.0 ± 2.4	43.5 ± 1.0^b^	42.5 ± 5.7	40.6 ± 2.8
LBWR	0.05 ± 0.01	0.09 ± 0.01^b^	0.10 ± 0.01	0.10 ± 0.01	0.05 ± 0.00	0.05 ± 0.00	0.12 ± 0.01	0.09 ± 0.01
Fasting glucose, mmol/L	9.6 ± 1.2	9.3 ± 0.8	8.4 ± 0.3	8.3 ± 0.4	9.1 ± 0.4	9.7 ± 0.8	8.8 ± 1.2	9.1 ± 0.9
2 h glucose, mmol/L	21.9 ± 4.9	20.3 ± 3.6	23.5 ± 3.2	19.3 ± 1.8	15.3 ± 1.1	20.2 ± 3.5	15.1 ± 2.9	17.7 ± 5.2
GTT AUC, mmol/L·h	1604 ± 181	1047 ± 211	2519 ± 254	2164 ± 171	1349 ± 95	1700 ± 294	1709 ± 190	2248 ± 460
AST, IU/L	171 ± 71	482 ± 158	427 ± 73	359 ± 91	151 ± 23	125 ± 25	231 ± 44	265 ± 46
ALT, IU/L	140 ± 88	352 ± 116	303 ± 38	336 ± 37	86 ± 21	60 ± 13	202 ± 62	156 ± 41
AST/ALT ratio	1.97 ± 0.4	1.45 ± 0.1	1.39 ± 0.2	0.72 ± 0.2^b^	2.73 ± 0.3	2.33 ± 0.5	1.75 ± 0.8	2.6 ± 0.8
Circulating FFA, nmol/μl	0.46 ± 0.07	0.55 ± 0.06	0.57 ± 0.06	0.46 ± 0.04	0.50 ± 0.03	0.44 ± 0.06	0.48 ± 0.09	0.30 ± 0.07
Hepatic FFA, nmol/mg	1.90 ± 0.15	1.66 ± 0.07	1.81 ± 0.14	1.97 ± 0.14				
α-SMA, % positive area			2.26 ± 0.4	2.29 ± 0.1			1.64 ± 0.4	1.28 ± 0.14

Abbreviation: AUC, area under the curve.

a All data are expressed as mean ± SEM.

b *P* < .05 vs WT.

### Hepatic phenotype in 5αR1^−/−^ and 5αR2^−/−^ mice on NC

Animals fed NC had normal liver histology at 6 months. By 12 months, only a small number of animals had evidence of steatosis or lobular inflammation, and only 1 had evidence of mild (stage 1b) fibrosis. There were no significant differences in the composite NAS histology, hepatic triglyceride/FFA levels, or mRNA expression of genes involved in lipid metabolism in WT, 5αR1^−/−^, and 5αR2^−/−^ mice (data not shown).

### Hepatic phenotype in 5αR1^−/−^ and 5αR2^−/−^ mice on the ALIOS diet

#### ALIOS diet for 6 months

**Figure 2.**
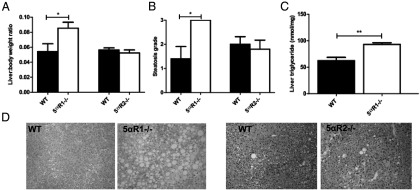
A–C, 5αR1^−/−^ mice fed the ALIOS diet for 6 months have increased LBWR (mean ± SEM) (A), histological steatosis grade (median) (B), and hepatic triglyceride quantification (mean ± SEM) (C) in comparison with WT and 5αR2^−/−^ mice. D, Representative H&E sections. *, *P* < .05; **, *P* < .005.

Liver to body weight ratio (LBWR) was higher in 5αR1^−/−^ but not 5αR2^−/−^ mice in comparison with WT controls on the ALIOS diet (Figure 2A and Table 2). In addition, histological Kleiner steatosis grade was greater in 5αR1^−/−^ mice (median 3 vs 1, *P* < .05), with the maximal steatosis grade 3 seen in all 5αR1^−/−^ mice but in only 20% of WT mice (Figure 2B). Formal hepatic triglyceride quantification confirmed increased hepatic lipid content in 5αR1^−/−^ compared with WT ALIOS mice (mean 93.1 ± 3.2 vs 62.4 ± 6.2 nmol/mg, *P* < .005, Figure 2C). Representative H&E-stained sections are shown in Figure 2D. Circulating and hepatic FFA levels were not different in WT compared with 5αR1^−/−^ or 5αR2^−/−^ mice (Table 2). At 6 months, serum alanine aminotransferase (ALT) was highest in the 5αR1^−/−^ mice, but this failed to reach statistical significance (352 ± 116 vs 140 ± 88 IU/L, *P* = .18) (Table 2).

**Figure 3.**
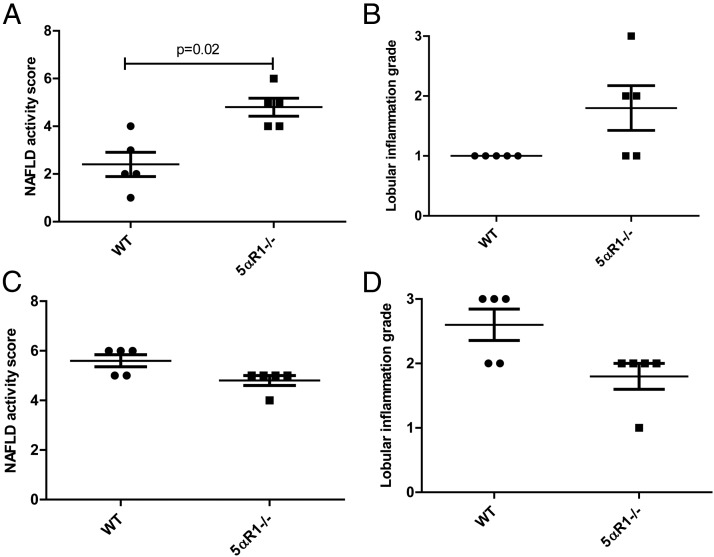
A and B, NAS is increased in 5αR1^−/−^ mice fed the ALIOS diet for 6 months (A) but without a significant change in lobular inflammation (B). C and D, There was no difference in the histological severity of NAFLD comparing WT and 5αR1^−/−^ mice after 12 months of the ALIOS diet.

**Figure 4.**
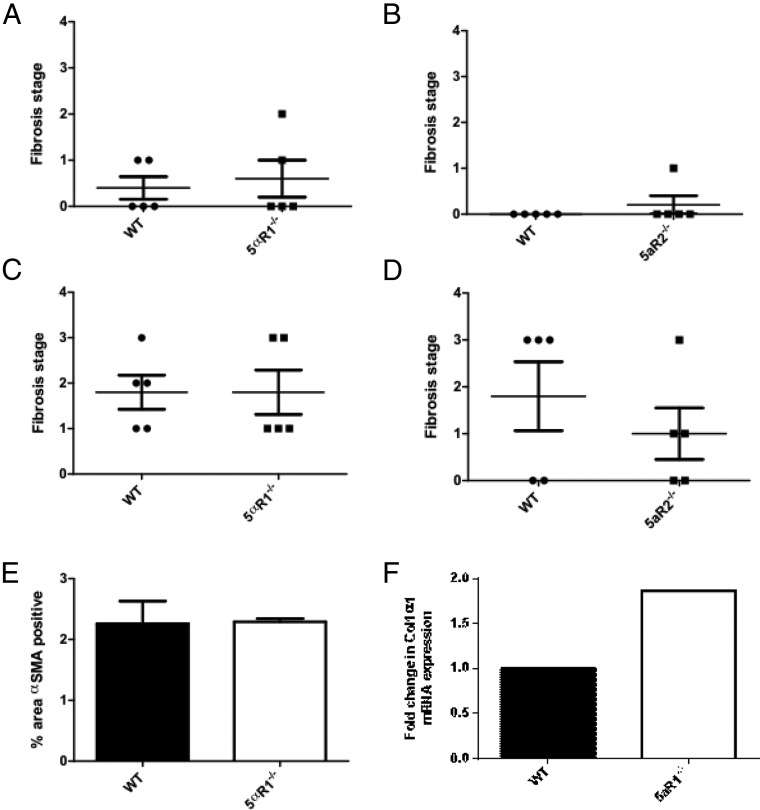
A and B, Fibrosis was infrequent at 6 months with mild stage 1c (periportal) or stage 2 fibrosis present in only 5 of 20 ALIOS-fed mice. C and D, At 12 months, fibrosis was present in 16 of 20 WT mice, with 7 of 20 displaying bridging (stage 3) fibrosis. The remainder had either portal/periportal (stage 1c) or portal/periportal and perisinusoidal (stage 2) fibrosis. There were no significant differences between WT and 5αR1^−/−^ or 5αR2^−/−^ mice. E and F, α-SMA expression (E) and *Col1*α*1* mRNA levels (F) were also similar in all groups of animals that had been fed the ALIOS diet for 12 months.

Histological NAS was higher in 5αR1^−/−^ mice compared with WT mice (median 5 vs 2, *P* < .05) (Figure 3A) at 6 months. No difference was seen in 5αR2^−/−^ mice (median 3 vs 2). The degree of lobular inflammation was also higher in the 5αR1^−/−^ mice but did not reach statistical significance (Figure 3B). Distinction of ballooned hepatocytes from the frequently extensive microvesicular steatosis made assessment of this histological feature difficult. Although occasional cells with a ballooned appearance were initially thought to be present in H&E-stained sections from ALIOS mice at both 6 and 12 months, no definite Mallory-Denk bodies were identified by immunostaining for ubiquitin or K18 (data not shown). For all animals, the ballooning component of the NAS was thus scored as 0. Fibrosis was infrequent at 6 months with mild stage 1c (periportal) or stage 2 fibrosis present in only 5 of the ALIOS-fed mice; there were no differences between WT and 5αR1^−/−^ or 5αR2^−/−^ mice (Figure 4, A and B) No neoplastic hepatocellular lesions were observed in any animals at 6 months.

**Figure 5.**
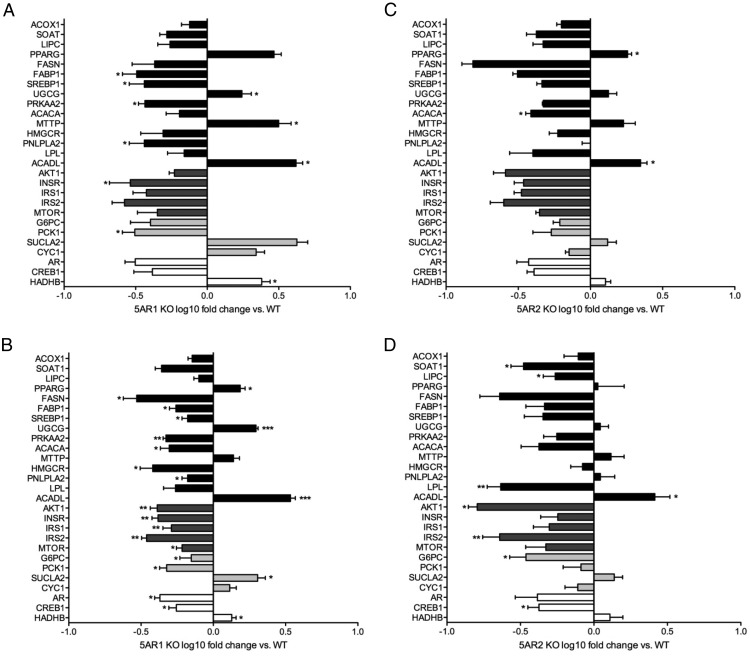
Hepatic gene expression measured using low-density real-time PCR-based assays in 5αR1^−/−^ (A and B) and 5αR2^−/−^ (C and D) mice fed an ALIOS diet for 6 (A and C) or 12 months (B and D) and expressed as fold change relative to WT mice. *, *P* < .05; **, *P* < .01.

In the 5αR1^−/−^ mice, the mRNA expression of many genes involved in insulin signaling was decreased, although only insulin receptor expression decreased significantly (0.3-fold, *P* < .05). Interestingly, the regulation of lipid metabolism genes was mixed with decreased expression of genes regulating both synthesis (*SREBP* 0.4-fold, *P* < .05) and mobilization (*PNLPLA2*, 0.4-fold, *P* > .05) (Figure 5A). Complete gene expression data are presented in Supplemental Table 2. Changes in the 5αR2^−/−^ animals were less marked with no regulation of insulin signaling cascade genes, *SREBP1* or *PNLPLA2*. However, *ACACA* expression decreased (0.5-fold, *P* < .05) (Figure 5B).

#### ALIOS diet for 12 months

After 12 months of the ALIOS diet, grade 3 steatosis was observed in 90% of WT mice. Similarly, lobular inflammation was present in 95%, with no differences in overall inflammation between WT, 5αR1^−/−^, and 5αR2^−/−^ mice. The NASs were also not different after 12 months on the ALIOS diet (Figure 3, C and D). Consistent with the histological findings, there were no significant differences between WT, 5αR1^−/−^, and 5αR2^−/−^ mice in serum aspartate aminotransferase (AST), ALT, and hepatic TNFα mRNA levels (Tables 2 and 3).

Fibrosis was present in 16 of 20 (80%) WT mice, with 7 of 20 (35%) displaying bridging (stage 3) fibrosis. The remainder had either portal/periportal (stage 1c) or portal/periportal and perisinusoidal (stage 2) fibrosis. There were no significant differences in 5αR1^−/−^ and 5αR2^−/−^ mice, and consistent with this, α-SMA expression and *COL1A1* mRNA levels were similar in all groups of animals that had been fed the ALIOS diet for 12 months (Figure 4, C–F, and Tables 2 and 3).

In 5αR1^−/−^ animals, there was significant downregulation in mRNA expression of many of the components of the insulin signaling cascade combined with a similar pattern in genes regulating lipid metabolism (Figure 5C and Supplemental Table 2). Observations were similar (although less marked) in 5αR2^−/−^ mice with decreased expression of lipoprotein lipase, *AKT1*, and *IRS2* (all *P* < .01) (Figure 5D and Supplemental Table 2). In the 5αR2^−/−^ animals, there was no compensatory increase in 5αR1 mRNA expression either at baseline or after the ALIOS diet (Supplemental Table 2).

### 5αR and hepatocellular neoplasia

After 12 months of the ALIOS diet, focal hepatocellular lesions suggestive of well-differentiated HCC were evident in 60% of WT ALIOS-fed mice but in only 20% (1 of 5) of 5aR2^−/−^ and 0% (0 of 5) of 5αR1^−/−^ mice (*P* < .05 vs WT). Histological analysis of the lesions identified in these mice demonstrated features consistent with HCC with an increased nuclear to cytoplasmic ratio, nuclear crowding, mitoses, fatty sparing, and loss of the normal reticulin fiber pattern, a characteristic feature of human HCC. The NAS in the background livers of these animals was ≥5 in all cases, consistent with the presence of NASH, and associated with F3 fibrosis in 67% (4 of 6) of animals that developed HCC. Androgen receptor was expressed in both the tumor and adjacent normal liver tissue with no apparent difference in protein level (data not shown). There were no differences in hepatocyte proliferation, as measured by the number of Ki67^+^ cells (33 ± 8 vs 33 ± 7, WT vs 5αR1^−/−^) seen per field of view. Pan-CK and SOX9 are markers of stem cell activation, which have been implicated in tumor pathogenesis. There were no differences in either SOX9 (Supplemental Figure 1, A–D) or pan-CK (Supplemental Figure 1, E–H) expression comparing 5αR1^−/−^ or 5αR2^−/−^ with WT animals.

### Contribution of androgens and GCs to hepatic steatosis

**Figure 6.**
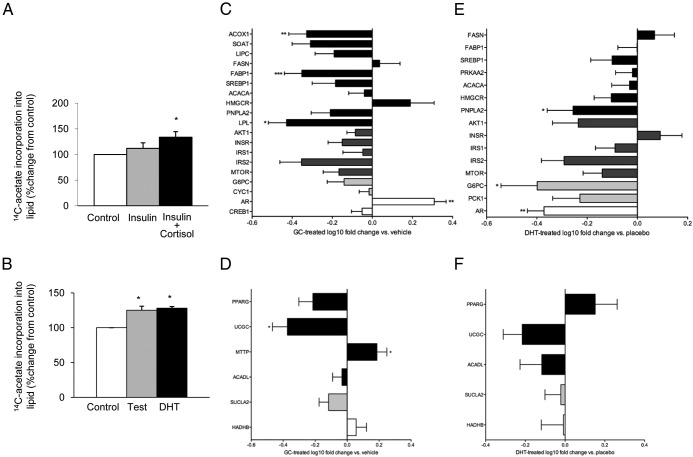
A and B, Cortisol (250nM, 24 hours) in combination with insulin (5nM) (A) and both testosterone (50nM, 24 hours) and DHT (10nM, 24 hours) (B) increase lipogenesis in human C3A human hepatoma cells. *, *P* < .05 vs control. C–F, Hepatic gene expression measured using low-density real-time PCR-based assays in corticosterone (C and D) and DHT-treated (E and F) mice. Data are expressed as fold change relative to placebo-treated animals. *, *P* < .05; **, *P* < .01. C and E, Genes that were negatively regulated in 5αR1^−/−^ mice fed an ALIOS diet for 12 months. D and F, genes that were positively regulated (see Figure 5).

Insulin had only a modest effect in C3A cells to stimulate lipogenesis, and this failed to reach statistical significance (111% ± 11%, *P* > .05). However, cortisol, together with insulin, significantly increased lipid accumulation (135% ± 11%, *P* < .05) (Figure 6A). Interestingly, both testosterone and DHT stimulated lipogenesis in vitro (testosterone, 125% ± 6%, *P* < .05; DHT, 128% ± 3%, *P* < .05) (Figure 6B).

Manipulation of 5αR has the potential to alter metabolic phenotype through increased GC availability and/or DHT deficiency. To try and identify which mechanism may be most important, hepatic gene expression profiles were compared in mice treated with either GC (corticosterone) or DHT. Corticosterone treatment regulated 18 of 25 genes (72%) in a similar pattern to that observed in 5αR1^−/−^ animals fed an ALIOS diet for 12 months (Figure 6, C and D). However, 14 of 20 genes (70%) were also regulated in a similar pattern to that observed in 5αR1^−/−^ mice in DHT-treated animals (Figure 6, E and F). These data suggest that the gene expression profiles in 5αR1^−/−^ mice more closely resemble GC excess rather than what would have been predicted in an androgen (DHT)-deficient model.

## Discussion

5αR1 and -2 are highly expressed in human liver (5αR1 only in rodent) and are known to regulate both exposure of androgen and GC, which have also been shown to regulate lipid metabolism, and therefore, this may suggest a role for 5αR activity in the regulation of metabolic phenotype. Our data have shown that 5αR1 protein levels increase with severity of NAFLD in humans and that in a rodent model, deletion of 5αR1 accelerates aspects of NAFLD progression. Notably, 5αR1 deletion protects mice from the development of HCC.

We and others have previously demonstrated increased 5αR activity in patients with insulin resistance (18) and NAFLD (19, 20, 30) as measured by urinary steroid metabolite analysis, and have proposed that this may represent an attempt to preserve liver phenotype by decreasing local GC availability. Endorsing this hypothesis, using immunohistochemistry, we have demonstrated increasing 5αR1 protein levels in human liver with histological progression of NASH, which correlated with the presence of lobular inflammation; 5αR2 protein levels were unchanged across the NAFLD spectrum.

5αR1^−/−^ mice fed the ALIOS diet had a significantly greater LBWR than WT mice, with histological steatosis and increased biochemical triglyceride accumulation at 6 months. By 12 months of the ALIOS diet, the impact of 5αR1^−/−^ was lost. This is likely to reflect the severity of the intervention, in that by 12 months, almost all animals had developed severe liver disease and therefore, the ability to detect a worsening of phenotype is lost. It is possible that a less aggressive intervention may have been able to demonstrate a more severe worsening of phenotype as a consequence of 5αR1 deletion. Importantly, 5αR2^−/−^ mice were not different from WT mice fed the ALIOS diet. This may reflect the lack of 5αR2 expression in the rodent (but not human) liver (15) and in addition suggests that extrahepatic 5αR2 does not contribute significantly to the development of NAFLD in rodents.

5αR isoforms are crucial in limiting GC availability and augmenting androgen action, and it is plausible that either (or both) of these mechanisms may underpin the observations that we have made. Lipid homeostasis within hepatocytes represents a balance between lipogenesis and utilization (principally β-oxidation and packaging into very-low-density lipoprotein). FFAs for lipid synthesis are available from the diet, lipolysis of triglyceride within adipose tissue, and de novo lipogenesis (DNL) within the liver (31). However, the main contributors to excessive hepatic fat accumulation in patients with NAFLD are increased FFA mobilization from adipose tissue and enhanced hepatic DNL (32). GCs drive lipogenesis and limit β-oxidation that may contribute to lipid accumulation (33, 34). In leptin-resistant rodents, selective hepatic GC receptor knockdown ameliorates fat accumulation within the liver, decreasing fatty acid uptake and increasing *ACC2* and *CPT1* expression (35). The patterns of gene expression that we observed were mixed. In 5αR1^−/−^ but not 5αR2^−/−^ mice, mRNA expression changes were consistent with the development of insulin resistance, with decreased insulin receptor expression, and this may have contributed to the observed hepatic phenotype. However, genes involved in both the synthesis and mobilization of lipid were downregulated. Many of these are posttranscriptionally regulated, and it is possible that the observed changes in mRNA expression may not translate to functional activity; a limitation of our study is that we did not examine the regulation of these enzymes at the protein level.

GCs can potentiate the action of insulin to promote lipogenesis within hepatocytes (33). Decreased GC clearance in 5αR1^−/−^ mice may therefore enhance the synergistic action of GCs to work with insulin to drive hepatic lipid accumulation. However, in addition, they have important anti-inflammatory actions, and their adverse effects on lipid mobilization and DNL could be counterbalanced by their anti-inflammatory action. We observed only a modest increase in lobular inflammation in the 5αR1^−/−^ mice at 6 months that was not apparent at 12 months, and it is possible that the inflammatory response at this time point may have been partially ameliorated by increased local GC availability. Interestingly, serum AST levels were lower in the 5αR1^−/−^ mice, although this did not reach statistical significance, and it is possible that this could reflect decreased inflammation in the face of increased lipid accumulation. In further support of this hypothesis, the combination of high-fat diet and exogenous GC treatment has a synergistic effect upon the development of steatosis and liver injury in rats, without an accompanying increase in inflammation (36).

Although we were unable to measure testosterone and DHT levels in our mice, male 5αR-knockout mice have previously been shown to have decreased circulating DHT levels (37), although in male 5αR1^−/−^ mice, circulating testosterone and DHT levels have been reported as similar to WT animals (38). Emerging evidence suggests that low levels of circulating androgens may be associated with NAFLD (12, 13). Hepatic AR-knockout mice fed a high-fat diet develop hepatic steatosis and insulin resistance (39), suggesting that decreased hepatic androgen availability in 5αR1^−/−^ mice may contribute to increased steatosis. The impact of androgens upon the inflammatory process is not clear, and the evidence from the literature is conflicting (40–44). Importantly, we have examined the hepatic phenotype only in male mice, and it is possible that observations may be different in female mice.

Our in vitro data suggest that exposure to both GC and androgen excess can drive lipogenesis. This adds weight to the argument that the impact of 5αR1 deletion to promote hepatic steatosis is driven largely by impaired GC clearance rather than decreased DHT generation. Furthermore, in the C3A cell line, both testosterone and DHT were equally able to stimulate lipogenesis, perhaps negating a specific role of 5αR1 in this regard. Further endorsing the role of GCs, the hepatic gene expression profiles that we observed in the 5αR1^−/−^ mice fed the ALIOS diet for 12 months more closely resembled that associated with corticosterone treatment and were actually similar to those seen in DHT-treated (as opposed to DHT-deficient) animals. One important caveat is that the DHT treatment was performed in female mice, and it is possible that there could be sexually dimorphic responses in response to androgen treatment.

In our studies, we have not replaced DHT in 5αR1^−/−^ mice to see whether this is able to reverse or ameliorate the hepatic phenotype that we have observed. They retain expression of 5αR2 (see Supplemental Table 2), circulating DHT levels have been shown to be similar to WT animals in previous studies (38), and accurate and reliable tissue-specific measures of hormone levels are technically challenging. Without the ability to accurately titrate intracellular hormone levels, DHT replacement similar to what we used in WT animals is likely therefore to result in circulating androgen excess.

A notable finding in this study was that 5αR1^−/−^ appears to exert a protective effect against the development of HCC. HCC has a higher incidence in males than females (45, 46), and both serum and intracellular DHT to testosterone ratios are increased in patients with HCC compared with those with cirrhosis alone or normal controls (47). DHT enhances HCC cell proliferation in an AR-dependent manner (48), and the regression of HCC in response to AR antagonism is related to the reduction in circulating DHT levels (49). AR expression was observed in all of the mouse livers affected by HCC in our study (data not shown), although this is not a universal finding in the published literature (50, 51). It is plausible therefore that 5αR1^−/−^ mice are protected from the development of HCC by decreased local androgen availability. The nonsteroidal, noncompetitive 5αR inhibitor FK143 has been used in a rodent HCC model (52), but studies in humans have not been performed. Tumor number, volume, and proliferative activity of HCC were significantly decreased alongside reductions in circulating DHT levels (52), and these published data endorse the observations made in our study. The activation of hepatic stem cells in response to chronic metabolic injury is well described with increasing evidence suggesting the accumulation of hepatic stem cells may increase risk of, or directly contribute to, the development of HCC. We therefore objectively quantified the activation of the hepatic stem cell niche to determine any effect on hepatocarcinogenesis. In all genotypes, hepatic stem cells were seen to accumulate in response to increasing duration of the ALIOS diet; however, no significant difference between WT and 5αR1^−/−^ or 5αR2^−/−^ mice was observed, suggesting that the protective effect of 5αR knockout may be mediated through an alternative mechanism.

In conclusion, these data support a critical role of 5αR1 in the evolution of NAFLD and a putative role in the development of HCC. Limited DHT availability as well as decreased GC clearance may both contribute to increased steatosis in 5αR1^−/−^ mice. Extending these observations, more clinical studies are needed to determine the impact of manipulation of 5αR activity upon metabolic phenotype, lipid metabolism, and the progression of NAFLD in humans.
